# Transcriptome sequencing reveals *CHD1* as a novel fusion partner of *RUNX1* in acute myeloid leukemia with t(5;21)(q21;q22)

**DOI:** 10.1186/s12943-015-0353-x

**Published:** 2015-04-11

**Authors:** Hong Yao, Jinlan Pan, Chunxiao Wu, Hongjie Shen, Jundan Xie, Qinrong Wang, Lijun Wen, Qian Wang, Liang Ma, Lili Wu, Nana Ping, Yun Zhao, Aining Sun, Suning Chen

**Affiliations:** Jiangsu Institute of Hematology, Key Laboratory of Thrombosis and Hemostasis of Ministry of Health, The First Affiliated Hospital of Soochow University, Suzhou, P.R. China; Collaborative Innovation Center of Hematology, Soochow University, Suzhou, P.R. China; Cyrus Tang Hematology Center, Jiangsu Institute of Hematology, the First Affiliated Hospital, Soochow University, Suzhou, P.R. China

**Keywords:** RUNX1, CHD1, Fusion gene, Acute myeloid leukemia

## Abstract

**Background:**

RUNX1/AML1, which is a Runt family transcription factor critical for normal hematopoiesis, is frequently mutated or translocated in a broad spectrum of hematopoietic malignancies.

**Findings:**

We describe here the case of a 54-year-old female developed acute myeloid leukemia with a t(5;21)(q21;q22). Transcriptome sequencing identified the chromodomain-helicase-DNA-binding protein 1 gene, CHD1, as a novel partner gene of RUNX1. Furthermore, the patient was found to harbor FLT3-ITD mutation, which might collaborated with CHD1-RUNX1 in the development of acute myeloid leukemia.

**Conclusions:**

We have identified CHD1 as the RUNX1 fusion partner in acute myeloid leukemia with t(5;21)(q21;q22).

**Electronic supplementary material:**

The online version of this article (doi:10.1186/s12943-015-0353-x) contains supplementary material, which is available to authorized users.

## Findings

The RUNX1 (previously AML1) gene encodes a DNA binding subunit of the core binding factor (CBF), which is critical for normal hematopoiesis. It is reported that *RUNX1* frequently mutated or translocated with at least 61 different chromosomal loci in a broad spectrum of hematopoietic malignancies. To date, more than 20 distinct RUNX1 gene fusions have been reported in a variety of hematologic malignancies [1-4]. However, about half of RUNX1 translocations remain uncharacterized at the molecular level. Identification of those unknown fusion partners of RUNX1 will provide more clues about the molecular and pathogenic mechanisms of these translocations. Recently, whole transcriptome sequencing (also known as RNA-Sequencing, RNA-seq) has been shown as an efficient tool to identify uncharacterized fusion genes [5]. We describe here the identification of a novel fusion gene involving *RUNX1* by case of a 54-year-old female developed acute myeloid leukemia with a t(5;21)(q21;q22) by transcriptome sequencing.

A 54-year-old female was admitted to our hospital in January 2011 because of fever and fatigue. Examination of peripheral blood indicated a platelet count of 103 × 10^9^/L, hemoglobin level of 60 g/L, and a white blood cell count of 9.37 × 10^9^/L with 22% circulating blasts. Bone marrow (BM) was hypercellular with 87.5%. Flow cytometry (FCM) immunophenotyping analysis showed positivity for CD34, CD14, CD13, CD33, CD117, CD15, CD11b and HLA-DR, as well as negativity for CD19, CD10, CD22, CD20, CD7, CD2, CD5 and CD3. The patient’s clinical picture was consistent with a diagnosis of AML-M4 according to the FAB classification, and AML not otherwise specified, acute myelomonocytic leukemia according to the World Health Organization (WHO) classification [6]. She was treated with induction chemotherapy of the IA regimen, including idarubicin and cytosine arabinoside. She achieved complete remission (CR) and received several courses of consolidation chemotherapy. However, her leukemia relapsed in April 2012. She was refractory to several courses of intensive combination chemotherapy and died in April 2013.

**Figure 1:**
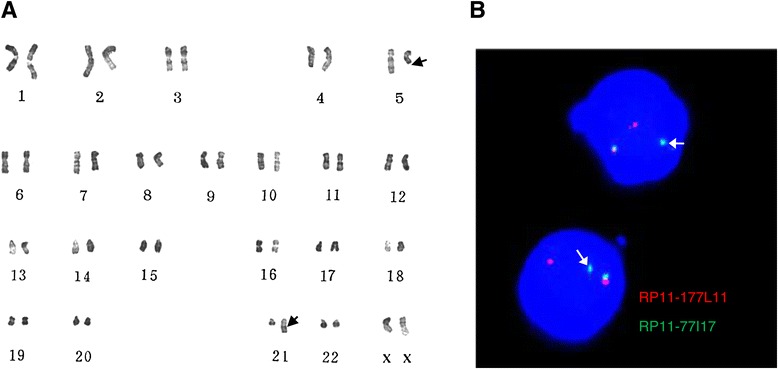
Cytogenetic and fluorescence in situ hybridization analyses of the patient. The bone marrow cells were cultured for 24 h and were analyzed for karyotyping and fluorescence in situ hybridization (FISH) with standard procedures. Clonal karyotypic abnormalities were described according to the International System for Human Cytogenetic Nomenclature (ISCN, 2009). **A)** Karyotype was analyzed on R-banded metaphases showing a t(5;21)(q21;q22), suggesting the involvement of the RUNX1 gene located at 21q22. **B)** FISH analysis of the rearrangement of RUNX1 by using BAC probes RP11-177 L11 (red) and RP11-77I17 (green).The separated red and green signals indicated a translocation involving RUNX1 gene.

The BM cells of this patient at presentation showed an karyotype of 46,XX,t(5;21)(q21;q22) (Figure 1A). RT-PCR analysis failed to detect *RUNX1-RUNX1T1* fusion transcripts from the bone marrow cells. For detection of RUNX1 rearrangements, dual color fluorescence in situ hybridization (FISH) experiments were performed with two contiguous BAC clones: RP11-177 L11, labeled with Spectrum Red-dUTP, and RP11-77I17, labeled with Spectrum Green-dUTP. FISH analysis showed one yellow signal corresponding to an intact *RUNX1* gene, one separated red signal, and one separated green signal indicating a translocation involving RUNX1 gene (Figure 1B). Thus, 3’-rapid amplification of cDNA ends (3’RACE) and RT-PCR were performed to investigate the fusion partner of RUNX1 and failed to detect positive results.

**Figure 2:**
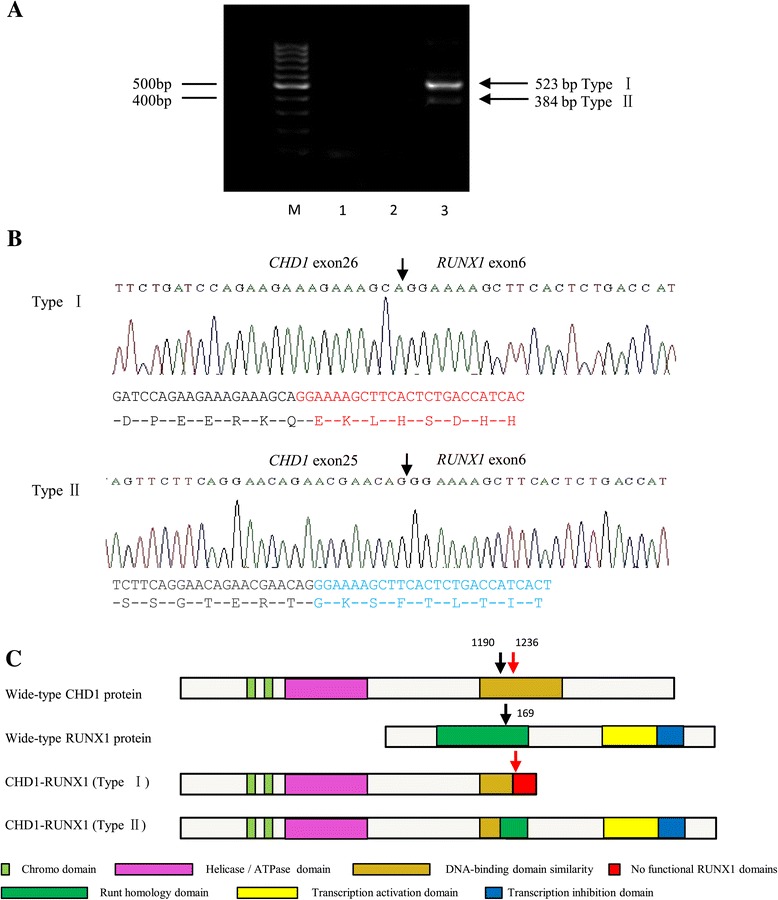
Characterization of the CHD1-RUNX1 fusion. **A)** RT-PCR confirmation for the CHD1-RUNX1 fusion. Lane M: 100 bp ladder; Lane 1: reagent control; Lane 2: negative control from a normal individual; Lane 3: CHD1-RUNX1 transcripts (523 bp and 384 bp) were detected in the patient discussed here. **B)** Sequencing analysis revealed two variant fusion transcripts between CHD1 and RUNX1.TypeI was a fusion between exon 26 of CHD1 and exon 6 of RUNX1, Type II was a fusion between exon 25 of CHD1 and exon 6 of RUNX1.The arrows indicated the fusion junction between CHD1 and RUNX1, the arabic numbers (1190, 1236, 169) indicated the amino acids position. **C)** Schematic structures of chimeric fusion proteins. The TypeII fusion protein retained the RUNX1 inhibition domain, however, the TypeI fusion created a frameshift and stop codon in the RUNX1 region, which resulted in a truncated protein without functional RUNX1 domain.

For the identification of the novel fusion gene in this patient, we performed whole-transcriptome sequencing of blast cells. Transcriptome sequence data were generated by high-throughput RNA sequencing performed on the Illumina HiSeq 2000. Candidate fusions were verified by RT-PCR amplification and bi-directional Sanger sequencing. Bioinformatic evaluation of the transcriptional sequencing data revealed 1 novel fusion, CHD1-RUNX1, in the patient. RT-PCR using primers for CHD1-RUNX1 fusion transcripts detected two bands (523 bp and 384 bp) corresponding to CHD1-RUNX1 fusion transcripts in this patient, while a normal individual had a negative result (Figure 2A). Sequence analysis indicated that the 523 bp PCR fragment was the product of a fusion event between exon 26 of *CHD1* and exon 6 of *RUNX1* (TypeI) (Figure 2B). In addition, we found that the 384 bp PCR fragment was the product of a fusion event between exon 25 of *CHD1* and exon 6 of *RUNX1* (TypeII) (Figure 2B). This suggested that the *RUNX1* breakpoint was in intron 5 and had generated alternative fusion splice variants. The typeII fusion transcripts retained part of the “Runt homology domain” (RHD) as well as the whole Runx transcription activation and inhibition domain (TAD and TID). However, the typeI fusion led to a premature stop codon (TAG) forty-two amino acids downstream of the junction point. This out-of-frame fusion resulted in a truncated fusion protein presumably with nonfunctional RUNX1 domains (Figure 2C). For the detection of the reciprocal RUNX1-CHD1 transcript, a *RUNX1* sense primer and a *CHD1* antisense primer were used but no fusion transcript could be detected.

Internal tandem duplication (ITD) mutations of the FLT3 gene have been described in approximate 20-25% of AML [7]. In the present case, we identified an internal tandem mutation of the FLT3 gene (FLT3-ITD) by using Gene Scanning as previously described [8] (Additional file 1: Figure S1).

Most chimeric gene involving RUNX1 fuse the 5’ part of the RUNX1 gene with the 3’ part of the partner gene. These fusion proteins retain RHD domain of RUNX1 which is responsible for heterodimerization with the core-binding factor-β (CBF-β) and DNA binding, but loss the TAD and TID domains, such as RUNX1-RUNXT1, RUNX1-MECOM, and RUNX1-LPXN [9]. However, there are two chimeric genes, namely *ETV6-RUNX1* and *USP16-RUNX1,* which fuse the 5’ region of partner gene with 3’ region of *RUNX1*. ETV6-RUNX1 retains RHD, TAD and TID domains of RUNX1. However, USP16-RUNX1 does not retain the RHD and no putative chimeric protein seems to be encoded due to loss of the open-reading frame [10,11]. Notably, our study identify a novel fusion gene *CHD1-RUNX1*, which is generated by 5’ region of *CHD1* and 3’ region of *RUNX1*, retains the whole TAD and TID, and part of RHD. The incomplete RHD is likely to impair the DNA binding capacity of RUNX1 or its heterodimerization with CBF-β.

*CHD1* locates in 5q15 and encodes a protein composed of 1710 amino acids. CHD1 is a chromatin-remodeling enzyme that belongs to the chromodomain family of proteins that play an important role in transcriptional regulation and developmental processes [12]. It has been reported that CHD1 is involved in assembly, shifting and removal of nucleosomes from the DNA double helix to keep them in an open and transcriptionally active state [13]. Two research groups have reported independently that *CHD1* plays a tumor-suppressor role in prostate cancer [14,15]. However, the role of CHD1 in hematological malignancies remains unknown. By analyzing karyotypic results of over 6000 newly-diagnosed patients with acute leukemia admitted to our institute between January 1985 and February 2015, we detected t(5;21)(q21;q22) translocation in two AML patients. One was a 47-year-old male patient who was diagnosed with AML-M2 in April 1994. The other one (the present case, NO. 201100834) was a 54-year-old female diagnosed with AML-M4. We identified the CHD1-RUNX1 fusion transcript from the female case.

Animal models have revealed that *RUNX1*-related translocations or haploinsufficiency of *RUNX1* are necessary but not sufficient for leukemogenesis [16,17], which suggests the requirement for additional genetic lesion for the development of leukemia. Internal tandem duplications (ITDs) in the juxtamembrane (JM) domain of FLT3 that lead to constitutive kinase activation in AML are associated with higher early relapse rate and inferior overall survival in patients with normal karyotype [18-20]. Furthermore, *FLT3-ITD* could cooperate strongly in leukemia induction with a variety of leukemia-initiating gene fusions such as *AML1-ETO*, *MLL-AF9*, or *PML-RAR α* [17,21,22]. We found the present patient harboring the *FLT3-ITD* mutation which might cooperate with *CHD1-RUNX1* in the induction of AML.

Taken together, we have identified a novel *CHD1-RUNX1* fusion consistent with the described t(5;21)(q21;q22) in a female patient with de novo AML (M4). Its role in the pathogenesis of AML still requires extensive investigation.

## Additional file

Additional file 1: Figure S1. Genescan analysis of FLT3-ITDs using automated capillary gel electrophoresis. A) WT-FLT3 (328 bp) was shown in a normal individual. B) WT-FLT3 and FLT3-ITDs (358 bp) was shown in the present case.
