# Supplementary figures and images for: Transcriptome sequencing reveals CHD1 as a novel fusion partner of RUNX1 in acute myeloid leukemia with t(5;21)(q21;q22)
Source: Mol Cancer. 2015 Apr 11;14:81. doi: 10.1186/s12943-015-0353-x (PMC4486139; doi:10.1186/s12943-015-0353-x)

Figure S1

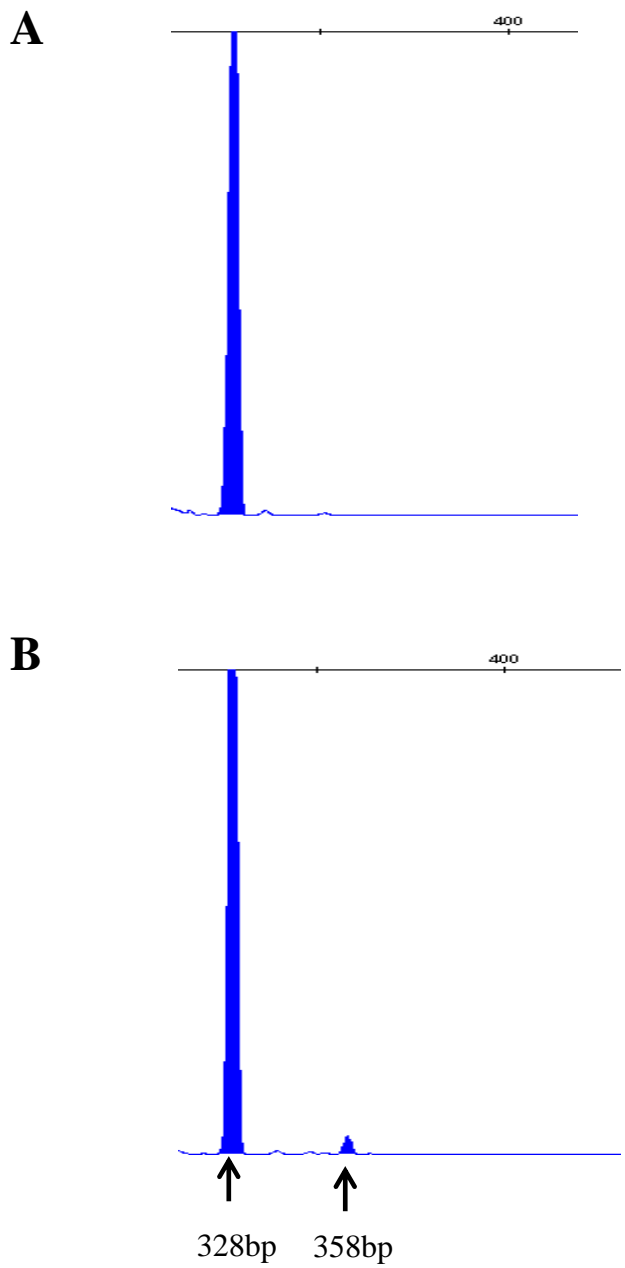

Supplement: Additional file 1: Figure S1. — Genescan analysis of FLT3-ITDs using automated capillary gel electrophoresis. A) WT-FLT3 (328 bp) was shown in a normal individual. B) WT-FLT3 and FLT3-ITDs (358 bp) was shown in the present case. [file 12943_2015_353_MOESM1_ESM.pdf]
